# Large-Scale Evaluation of Major Soluble Macromolecular Components of Fish Muscle from a Conventional ^1^H-NMR Spectral Database

**DOI:** 10.3390/molecules25081966

**Published:** 2020-04-23

**Authors:** Feifei Wei, Minoru Fukuchi, Kengo Ito, Kenji Sakata, Taiga Asakura, Yasuhiro Date, Jun Kikuchi

**Affiliations:** 1 RIKEN Center for Sustainable Resource Science, 1-7-22 Suehiro-cho, Tsurumi-ku, Yokohama 235-0045, Japan; kengo.ito@riken.jp (K.I.); kenji.sakata@riken.jp (K.S.); taiga.asakura@riken.jp (T.A.); yasuhiro.date@riken.jp (Y.D.); 2 Graduate School of Medical Life Science, Yokohama City University, 1-7-29 Suehirocho, Tsurumi-ku, Yokohama 230-0045, Japan; minoru.fukuchi.1@gmail.com; 3 Graduate School of Bioagricultural Sciences and School of Agricultural Sciences, Nagoya University, 1 Furo-cho, Chikusa-ku, Nagoya 464-8601, Japan

**Keywords:** NMR, macromolecules, fish muscle extracts, matrix decomposition

## Abstract

Conventional proton nuclear magnetic resonance (^1^H-NMR) has been widely used for identification and quantification of small molecular components in food. However, identification of major soluble macromolecular components from conventional ^1^H-NMR spectra is difficult. This is because the baseline appearance is masked by the dense and high-intensity signals from small molecular components present in the sample mixtures. In this study, we introduced an integrated analytical strategy based on the combination of additional measurement using a diffusion filter, covariation peak separation, and matrix decomposition in a small-scale training dataset. This strategy is aimed to extract signal profiles of soluble macromolecular components from conventional ^1^H-NMR spectral data in a large-scale dataset without the requirement of re-measurement. We applied this method to the conventional ^1^H-NMR spectra of water-soluble fish muscle extracts and investigated the distribution characteristics of fish diversity and muscle soluble macromolecular components, such as lipids and collagens. We identified a cluster of fish species with low content of lipids and high content of collagens in muscle, which showed great potential for the development of functional foods. Because this mechanical data processing method requires additional measurement of only a small-scale training dataset without special sample pretreatment, it should be immediately applicable to extract macromolecular signals from accumulated conventional ^1^H-NMR databases of other complex gelatinous mixtures in foods.

## 1. Introduction

There is growing interests focusing on seafood as a critical source of animal protein to meet the nutritional demands of a growing population within environmental limits. Notably, the rate of fish consumption has increased twice as rapidly as population growth since the 1960s, making fish as one of the most traded food commodities [1]. Nutraceutical products made from fish, such as fish lipids and oils, are one of the sources of vitamin D and omega-3 polyunsaturated fatty acids (n-3 PUFA), e.g., docosahexaenoic acid (DHA) and eicosapentaenoic acid (EPA), which, for instance, have been shown to be effective in reducing the risk of coronary heart disease [2]. In addition, fish scales are used in artificial pearls, isinglass, a form of collagen and gelatin, is prepared from the air bladders of certain species, and glue is made from fish offal [3]. Therefore, beside small molecule components [4,5], comprehensive characterization of the macromolecular components of fish such as collagen and gelatin could contribute to a greater understanding the metabolite diversity and function of fish.

Chromatography-and mass spectrometry-based proteomics and lipidomics analyses usually require a sample preparation step, whereas nuclear magnetic resonance (NMR) is an ideal and simple nondestructive approach to realize direct and comprehensive observation of complex mixtures with minimal sample preparation. NMR-based metabolomics has been widely applied in the profiling of small molecular components as well as macromolecular components, in combination with diffusion filter, in the food mixtures [6]. Nicolson et al. introduced a useful and beneficial methodology to extract composition profiles of complex matrix such as urine, plasma, serum and tissue extracts, using standard NMR pulse sequences, including 1D NOESY-presat, CPMG-presat, *J*-resolved, diffusion-edited and a single-pulse sequence [7]. Among these, diffusion-editing could be used to select mainly macromolecule-derived signals. However, in the metabolomics analysis of food mixtures, a large amount of conventional ^1^H-NMR spectral datasets have been measured and processed with only water suppression. These datasets are suffering from the limitation that the high intensity signals from small molecular components in the ^1^H-NMR spectra of complex mixtures mask the signals from soluble macromolecular components with baseline appearance due to the limitation of their solubility and concentration as well as their rapid relaxation mechanism and signal broadening [8].

This study is aimed to develop an integrated analytical strategy to dissect overlapping component ^1^H-NMR spectra and extract signals of soluble macromolecular components without the requirement of re-measurement. This strategy enables the reuse of the measured conventional ^1^H-NMR spectral data of food mixtures. A method using signals of pure reagents to separate ^1^H-NMR spectra for small molecular components has been previously developed [9]. However, this method is not applicable for signal separation of macromolecular components as appropriate internal standards are limited. Pure macromolecular reagents often have different molecular weights and solubility, and they lack complicated interactions compared with their natural state in the extremely complex matrices of biological samples. Thus, accurate extraction of their signals from the ^1^H-NMR spectra of complex mixtures is difficult. Currently, there are no efficient methods to extract signals of macromolecular components from conventional ^1^H-NMR spectral data of biological fluids without re-measurement using specific diffusion filters. Here, we introduced an integrated analytical strategy that combined a small scale of diffusion-edited measurement, covariation peak separation, and matrix decomposition to extract signal profiles of soluble macromolecular components from a large scale of pre-measured conventional ^1^H-NMR spectra of biofluids without the need for re-measurement. We applied this method to the conventional ^1^H-NMR spectra of water-soluble extracts of fish muscle and successfully analyzed the distribution characteristics of fish diversity and soluble major macromolecular components of muscle, such as lipids and collagens.

## 2. Results and Discussion

**Figure 1:**
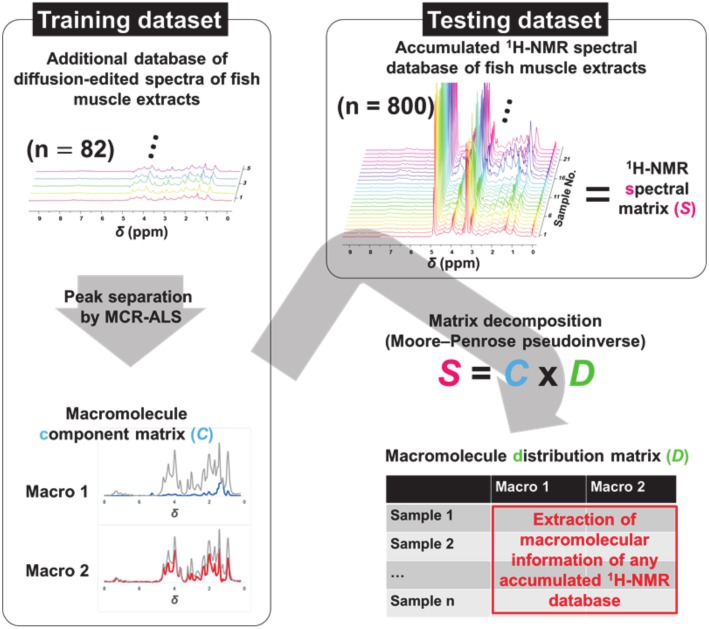
A schematic representation of the experimental design.

In this study, the two datasets shown in Figure 1 were used. One is the small-scale training dataset containing 82 diffusion-edited ^1^H-NMR spectra of fish muscle extracts. The NMR spectra of the small-scale dataset were digitized and peak separation was performed using multivariate curve resolution-alternating least squares (MCR–ALS) [10,11] to generate a macromolecular component matrix (Matrix C). The other dataset is the large-scale test data containing 800 conventional ^1^H-NMR spectra of fish muscle extracts (Matrix S). We assume that Matrix S is the product of matrix multiplication of Matrix C and another macromolecule distribution matrix (Matrix D). Therefore, the macromolecular signals could be extracted from the accumulated conventional ^1^H-NMR spectral data (Matrix S) based on the calculation of Matrix D.

**Figure 2:**
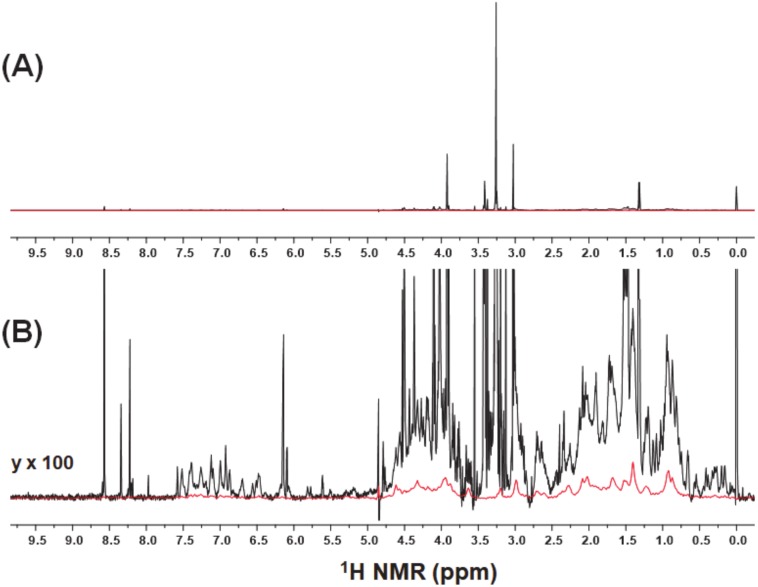
(**A**) Entire view and (**B**) Y-axis expansion of ^1^H-NMR spectra of fish muscle extracts with (in red) and without (in black) a diffusion filter.

A representative ^1^H-NMR spectrum of fish muscle extract was presented in Figure 2A. The conventional ^1^H-NMR spectra of water-soluble fish muscle extracts are dominated by high-intensity signals from small molecular metabolites [12,13,14,15]. In contrast, the broad signals from soluble macromolecular components close to the baseline were observed in the diffusion-edited spectra at 95% of the maximum magnetic field strength presented, while the signals from fast diffusion metabolites with low molecular weight were attenuated by diffusion filters (Figure 2B). In actual biological samples, the macromolecular components exist as complex mixtures of degradation products or fragments with different molecular weights, high structures, and molecular interactions, leading to signal broadening in ^1^H-NMR spectra.

To comprehensively identify the signals from macromolecular components, peak separation was performed on the measured diffusion-edited ^1^H-NMR spectra of fish muscle extracts. Since the MCR–ALS analysis based on a bilinear mode extracted the component spectra with Gaussian-shaped peak similar to the NMR spectra, the peak separation results of the MCR–ALS analysis were used in this study for further analysis.

**Figure 3:**
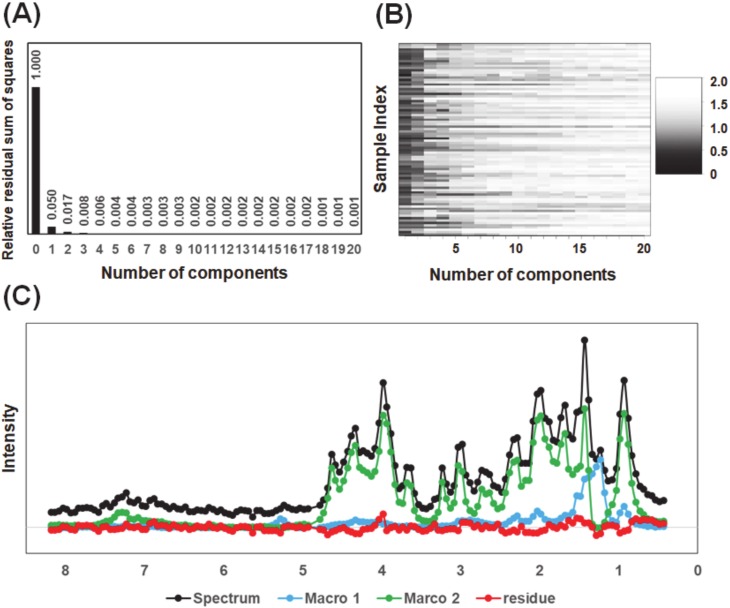
Peak separation of diffusion-edited ^1^H-NMR spectra of fish muscle extracts. (**A**) The relative residual sum of squares (RSS); (**B**) the Durbin-Watson (DW) color plot of multivariate curve resolution-alternating least-square (MCR–ALS) models; (**C**) the original diffusion-edited ^1^H-NMR spectrum (black), peaks of separated components (blue and green) and residue (red) generated by MCR–ALS model with the component number of 2.

Another important issue is to determine the number of components in mixtures of linear regression models. The clues to address this may be provided by the RSS and DW values [16]. The MCR–ALS models of 82 diffusion-edited ^1^H-NMR spectra of fish muscle extracts with input component number of 1–20 were established and the RSS and DW values of each model were calculated. As shown in Figure 3A, the RSS value of the model reduced sharply with increasing number of components from 0 (the data) to 1. The RSS value decreased to less than 2% when the number of components was 2, which indicated the explained sum of squares (ESS) has been more than 98% with a 2 component MCR-ALS model. No obvious decrease in the RSS value was observed until the number of components increased to 20. Compared with the pure reference spectra in the simulated dataset, it is difficult to distinguish signals from noise in the ^1^H-NMR spectra of the actual biological mixture. Similar results were also observed using the DW criterion (Figure 3B). Although some samples contain obvious noise even in the 20 component-model, most of the samples confirmed a turning point in DW value when the number of components increased from 2 to 3. Therefore, in the present study, two was chosen as the optimal number of components for the MCR–ALS analysis. Indeed, a representative example of peak separation of diffusion-edited ^1^H-NMR spectra of fish muscle extracts using two major components demonstrated that the separated peaks nearly completely represented the spectral profile of water-soluble macromolecular components (Figure 3C).

Consequently, peak annotation was performed to further understand the biological implication of these two major components. Compared with the pure macromolecular reagents, the water-soluble macromolecular components in an actual biological mixture are not considered to be pure substances. They are possibly complex mixtures of various degradation fragments with high solubility generated during the sample preparation process, making annotation of their separated peaks a challenge. The signal pattern of extracted component 1 (Macro 1) showed high similarity with the reference spectrum of fish oil [17,18]. KPi extraction and diffusion-edited spectrum measurement were performed on commercial fish oils in the same manner as that on fish muscles. As the results (Figure S1), there was a high similarity between the component Macro1 extracted by MCR-ALS and fish oil. For example, the typical methine signals of unsaturated fatty acids at approximately 5.4 ppm, protons due to glycerol at 4.1–4.3 ppm, as well as methylene and methyl protons due to unsaturated fatty acids coincide with the loading of Macro 1. Compared with the pure reagents, the signals of Macro1 generated from peak separation of the actual metabolic mixture showed a relatively lower signal resolution, which is likely due to the status of the lipids and its complicated interactions with other molecules in the complex mixtures. We propose that the signals of Macro 1 represent the changes of a small amount of lipids dissolved or emulsified in the KPi extraction process.

The signals of components 2 (Macro 2) showed a more complicated pattern. The broad peaks around 7.4 ppm indicated that Macro 2 contains polypeptide backbone N-H functional groups, which are probably because the higher structure of Macro 2 limited the H-D exchange rate between its backbone N-H functional groups and D_2_O in the solvent. Regarding the composition of fish muscle proteins, it is well known that the sarcoplasmic proteins account for 15–20% of the total fish muscle proteins, and the fibrous proteins account for approximately 60–80% of the total fish muscle proteins, while muscle fibrous proteins such as actomyosin are difficult to dissolve in water. Here, we found that the signals of Macro 2 showed a high similarity with the reference spectrum of collagen (Figure S1). Collagens, the most abundant components of the vertebrate extracellular matrix and connective tissues, play critical roles in the regulation of the functional and rheological properties of the flesh and the tensile strength of the muscles. The solubility of muscle collagen of fishes is known to be higher than that of mammals and is closely related with the storage conditions such as temperature and time period of fish and the type of collagens. In this study, the fish samples were quickly dissected and freeze-dried after thawing under a water flow and all the samples were prepared and measured under identical conditions. Therefore, the relative intensity of the major macromolecular components could be used to investigate the diversity of soluble collagen distribution in fish muscle. Compared with the reference spectrum of pure reagents, the major macromolecules mathematically identified in this study collectively provide valuable information on the characterization of water-soluble lipid and collagen contents and the composition of fish muscle extracts, which will serve as a measure to evaluate the biological diversity of marine ecosystems. As shown in Figure 3C, water-soluble collagens account for a large proportion of the macromolecular components of fish muscle. The differences between the measured reference spectra and the calculated results in Figure S1 can be considered due to the different existing states of macromolecules in pure and complex biofluid matrices.

**Figure 4:**
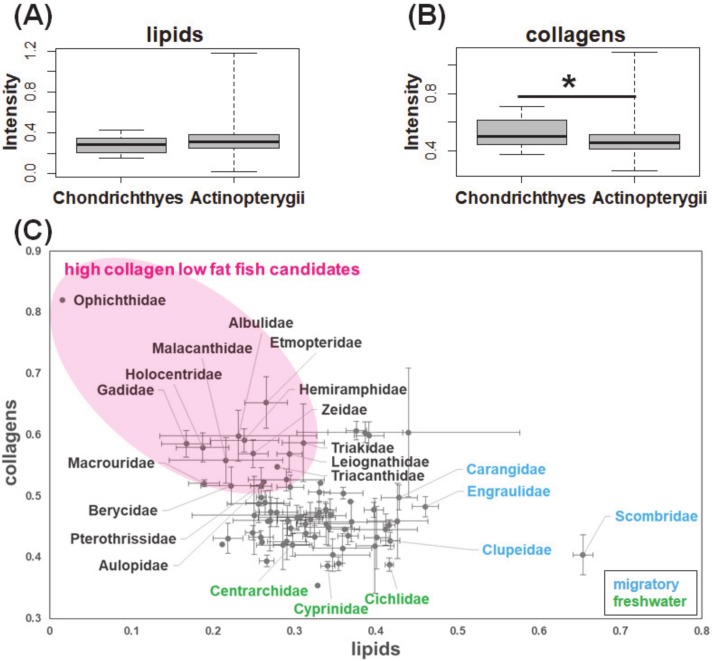
Macromolecular distribution in fish diversity: box plots of (**A**) lipids (*p*-value = 0.2156) and (**B**) collagens (*p*-value = 0.02159, * indicates a significant difference when *p*-value < 0.05) according to class, and (**C**) scatter plots according to family (mean ± standard error).

Finally, the loading of the above water-soluble major components extracted by the combination of diffusion-edited measurement and peak separation was used in the matrix decomposition of 800 conventional ^1^H-NMR spectra of fish muscle extracts. The basis for such an operation is that the conventional ^1^H-NMR without a diffusion filter also acquires signals from soluble macromolecular components, which are almost masked by the sharp and high-intensity signals from small molecular components. The advantage of application of standard loading for matrix decomposition on the entire ^1^H-NMR spectrum is that it could directly extract the peaks of interest derived from macromolecular components without the need to remove or filter out the high-intensity and complex signals from small molecular components. The distribution of the two water-soluble major macromolecular components in various fish classes was demonstrated in Figure 4. The box plot demonstrates that the two major macromolecular components of fish muscle extracts have different relative intensities in Actinopterygii and Chondrichthyes (Figure 4A,B). Compared with those in Chondrichthyes, the relative intensity of the lipid-like component Macro 1 was higher, while those of the collagen-like component Macro 2 was lower in Actinopterygii. This result is consistent with previous studies regarding the distribution of lipids and proteins in fish [19,20,21]. The muscle tissue of Chondrichthyes is characterized by higher collagen and lower fatty acids compared with those of Actinopterygii. Large amounts of lipid storage in the liver tissue, which are high in energy and low in density, are expected to assist white shark movement patterns [22]. In accordance with the report by Sato et al., our data suggested that the content of collagens is related to the flexibility of fish muscle [23].

The distribution of the two major macromolecular components according to fish families was summarized in Figure 4C. The lipid-like component Macro 1 has a high intensity in the migratory fish, such as Scombridae, Carangidae, Clupeidae, and Engraulidae, which is often used as a raw material for food industry products processed with fish oil. The collagen-like component Macro 2 has low intensity in the fresh-water fish such as Cyprinidae, Centrarchidae, and Cichlidae. Collectively, the present study investigated the chemical composition of lipids and collagens in fish muscles at a variety of diversity levels, including two classes and 209 species. As highlighted in Figure 4C, based on the matrix decomposition of the measured conventional ^1^H-NMR spectral data, we identified a cluster of fish species with low content of lipids and high content of collagens in muscle, which showed great potential for the development of functional foods.

## 3. Experimental

### 3.1. Materials and Sample Preparation

A total of 800 fish samples were sampled from June 2011 to February 2017 at multiple sites around Japan (Table S1) [4,24,25,26,27,28]. After dissection, the fish muscle samples were freeze-dried and crushed into powder. The powdered samples (18 mg) were mixed with 600 uL of KPi/D_2_O (D, 99.9%) buffer, and then metabolites were extracted at 65 °C for 15 min by following the method described in a previous study [5]. 4,4-Dimethyl-4-silapentane-1-sulfonate (DSS-d_6_, Wako, Osaka, Japan; diluted to a final concentration of 0.1%) was used as the internal reference, and its chemical shift was set to 0 ppm.

### 3.2. NMR Spectroscopy

The extracted samples were measured on an AVANCE II 700 spectrometer (Bruker BioSpin GmbH, Rheinstetten, Germany). The ^1^H-NMR spectra were observed using a Bruker standard pulse program “p3919gp” with solvent suppression by the WATERGATE sequence [29,30]. A 90° pulse angle was used, and the spectral parameters for the large-scale dataset were as follows: number of data points, 32 k; spectral width, 9804 Hz; acquisition time, 1.67 s; delay time, 2.5 s; and number of scans, 64. A Bruker standard pulse program “ledbpgp2s1d” sequence was used to observe the diffusion-edited spectra for the small-scale dataset (n = 82), a 90° pulse angle was used and the parameters were as follows: diffusion delay time (big delta; Δ), 300 ms; eddy current delay Te, 4.17 ms; gradient pulse width (little delta; δ), 3 ms; sine-shaped gradient, 40 G/cm (95% of the maximum magnetic field strength 48.15 G/cm); gradient recovery delay, 200 μs; spectral width, 10504 Hz; data points, 16 k; and number of scans, 16. This study is aimed to extract the relative distribution characteristics, rather than the absolute quantification of each component in the complex mixtures under identical experimental conditions. Therefore, a short *T*_1_ relaxation time, which is suitable for soluble macromolecules, was used in this study.

### 3.3. Data Processing

The raw NMR data were processed by Mnova (v.12.0.1, Mestrelab Research SL, Santiago de Compostela, Spain). After performing Fourier transform, auto phase adjustment, auto baseline correction, and chemical shift calibration, the spectral data of both small-scale training dataset and large-scale test dataset were reduced into 0.05 ppm spectral buckets from 0.45 to 8.20 ppm, and the data were saved as Excel files. The matrix size of the small- and large-scale datasets were 82 × 154 and 800 × 154, respectively.

Probabilistic quotient normalization (PQN) was performed on the small-scale dataset using R (v. 3.4.4). For metabolomics research, the integral normalization will be affected by strong metabolic changes and result in incorrectly scaled spectra, whereas the PQN method can avoid this problem based on the calculation of a most probable dilution factor [31]. Subsequently, peak separation was performed using the online server Peak Separation R (http://emar.riken.jp:3838/PKSP/3/), which was originally developed in our laboratory [32]. The MCR–ALS method was used to separate the macromolecular components in diffusion-edited spectral dataset. By MCR-ALS, an optimized bilinear relation between the experimental data, the concentrations and the pure spectra of the components is assumed iteratively by alternation least squares optimization, according to the generalized law of Lambert-Beer [33,34]. In matrix form, the model can be expressed as:

(1) $$X={\mathrm{AB}}^{T}+E$$

where X(i, j) is the spectral dataset of dimensions i samples by j NMR spectral buckets; A(i, k) and B^T^(k, j) are matrices which are related with the concentration profiles and pure spectra of the k components; E is the matrix of the residuals not explained by the chemical species in A and B. In the present study, i is 82 samples; j is 154 spectral buckets; k is determined by the modal residuals. Next, the pure spectral matrix B^T^ corresponding to the selected k was used as the macromolecular component Matrix C in the decomposition of the large-scale ^1^H-NMR spectral dataset to assume the concentrations.

The appropriate number of components was determined according to the values of residual sum of squares (RSS), and Durbin-Watson (DW). RSS is defined as a sum of squared residuals between the original and the reconstructed X for each model. The model corresponding to a minimal RSS is optimal. The DW criterion has been proposed as a measure of signal/noise ratio in signals; it tends to 0 when there is no noise in the signal and tends towards 2 if the signal contains only noise [35]. In order to determine the number of pure components k, the models were first established with a number of components from 1 to 20 and then the RSS and DW values were calculated for each model. As explained in detail in the Section 2, a component number of 2 was chosen as the optimal number of components for the MCR–ALS analysis in this study. Therefore, the matrix size of Matrix C after peak separation was 2 × 154.

After PQN, Matrix S was decomposed as the product of matrix multiplication of Matrix C and another distribution Matrix D using R. Finally, the distribution characteristics of fish diversity and muscle soluble macromolecular components were determined according to the distribution of the signal intensity in Matrix D.

## 4. Conclusions

The reference spectra of pure substances often have different molecular weights and solubility as well as interactions with other molecules. By contrast, the reference loading generated on the basis of the combination of diffusion-edited measurement and covariation peak separation in a small-scale training dataset is applicable for matrix decomposition to extract natural and accurate signal profiles of soluble macromolecular components in actual biological mixtures This strategy is useful for the analysis of a large-scale sample dataset without the need predetermined possible macromolecular components and additional NMR re-measurement. Therefore, it is expected to find immediate applicability in the extraction of macromolecular signals from accumulated conventional ^1^H-NMR databases of other biological fluids and complex polymer mixtures. Although it is easier to run a diffusion edited spectrum for a new analysis, this study provided an analytic strategy for reuse of the huge amount of previously measured conventional ^1^H-NMR spectral data to extract signal profiles of soluble macromolecular components. This will be particular important for the analysis of small and precious samples such as human clinical specimens. However, it should be noted that a training dataset will be required to determine the component matrix C for matrix decomposition on a large dataset of macromolecular mixtures with unknown composition. To address this limitation, further efforts need to be made to establish a ^1^H-NMR spectra database for soluble macromolecules of different biological fluids such as tissue extracts, blood, and urine.

## Supplementary Materials

The following are available online, Figure S1: DOSY spectra of authentic collagens and fish oils, Table S1: Sample list of fish used in the present study.
