# Supplementary material for: Large-Scale Evaluation of Major Soluble Macromolecular Components of Fish Muscle from a Conventional 1H-NMR Spectral Database
Source: Molecules. 2020 Apr 23;25(8):1966. doi: 10.3390/molecules25081966 (PMC7221887; doi:10.3390/molecules25081966)
Supplement: Supplementary file 1 [file molecules-25-01966-s001.zip › Figure S1.docx]

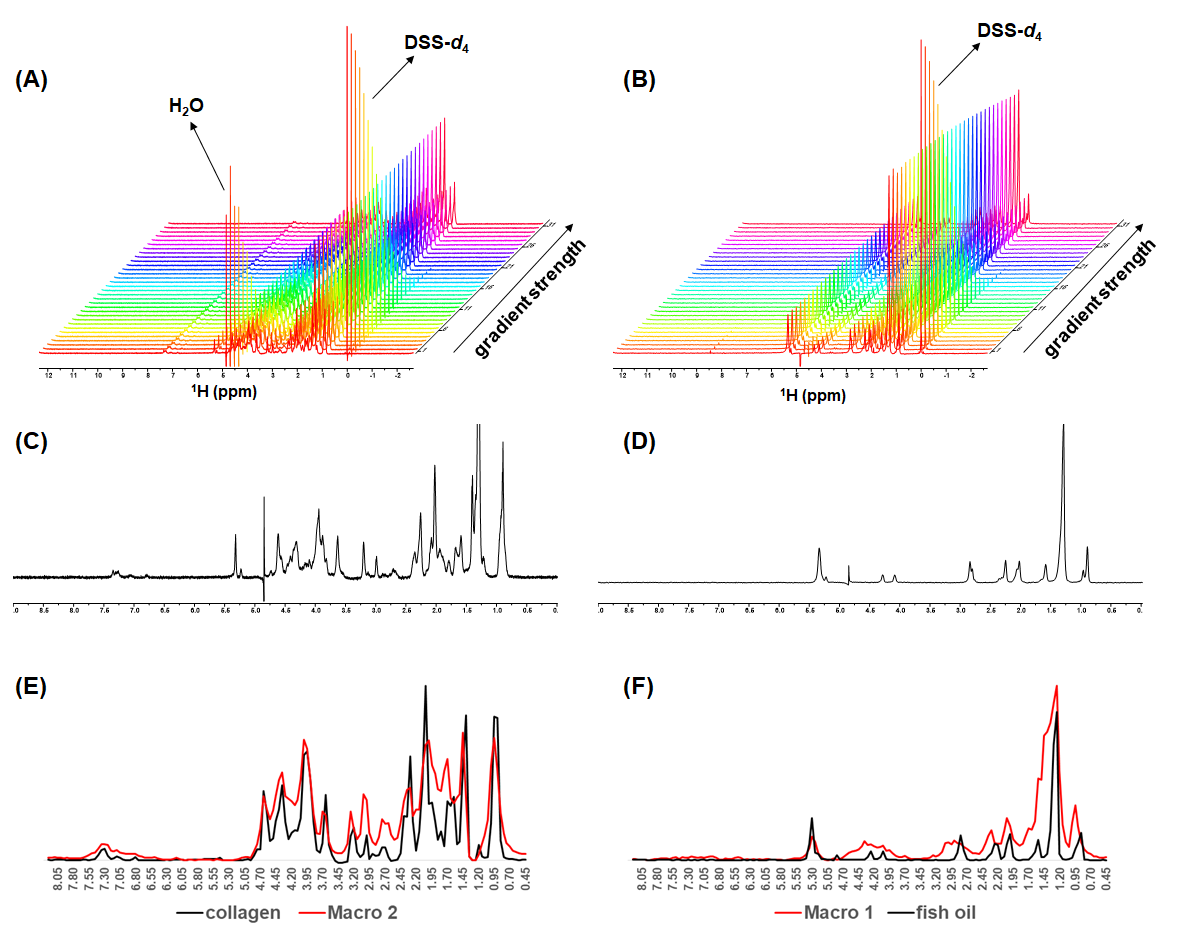


**Figure S1**. DOSY spectra of authentic **(A)** collagen from bovine achilles and **(B)** fish oils. The gradient strength is from 2% to 95% of the maximum magnetic field strength 48.15 G/cm; **(C)** and **(D)** are the slices of 95% of the maximum magnetic field strength of **(A)** and **(B)**, respectively; **(E)** and **(F)** are spectral data after binning (in black) and calculated components (in red).
